# A submission checklist to improve transparency and replicability of functional genomics data analyses

**DOI:** 10.1016/j.jbc.2026.111285

**Published:** 2026-03-24

**Authors:** Austin J. Hepperla, Ross C. Hardison, Roger J. Colbran, Brian D. Strahl, Jeremy M. Simon

**Affiliations:** 1 Department of Genetics, University of North Carolina at Chapel Hill, Chapel Hill, North Carolina, USA; 2 Bioinformatics and Analytics Research Collaborative (BARC), University of North Carolina at Chapel Hill, Chapel Hill, North Carolina, USA; 3 Department of Biochemistry and Molecular Biology, The Pennsylvania State University, University Park, Pennsylvania, USA; 4 Department of Molecular Physiology and Biophysics, Vanderbilt University School of Medicine - Basic Sciences, Nashville, Tennessee, USA; 5 Department of Biochemistry and Biophysics, University of North Carolina, Chapel Hill, North Carolina, USA; 6 Department of Data Science, Dana-Farber Cancer Institute, Boston, Massachusetts, USA; 7 Department of Biostatistics, Harvard T.H. Chan School of Public Health, Cambridge, Massachusetts, USA

The mission of the *Journal of Biological Chemistry* (JBC) is to publish science that is rigorous, reproducible, and useful to the community of molecular life scientists. That goal is only achievable when others can critically assess the work performed, reanalyze data when appropriate, and build upon the findings with confidence. To that end, the journal views its responsibility as ensuring that the data underlying published claims are readily accessible and that the mechanism for accessing those data is clearly provided.

An increasing number of JBC submissions include data from a variety of functional genomics experiments, including RNA-seq, DNA-seq (*e.g.* whole-genome and whole-exome sequencing), ChIP-seq, CUT&RUN, ATAC-seq, and many other high-throughput approaches in bulk or single cells, which are now routine across the molecular biosciences. These methods are powerful, but when data access, metadata, and analysis workflows are not clearly reported, the datasets and processing steps underlying key conclusions can be difficult to locate, evaluate, and interpret. This creates a recurring challenge for authors, reviewers, and readers, especially for end users of these data who may not be experts in the assays and analytical methods. Rigorous review and meaningful reuse depend on being able to find the underlying data and understand how they were generated and analyzed.

For genomics and functional genomics datasets, “data access” means more than a figure panel or a supplementary table. It means that reviewers as well as readers should be able to download both original and processed datasets to understand, at a basic level, how those data were generated and analyzed. It also means that key experimental variables and sample attributes—along with algorithms and/or code utilized for data processing, analysis, and biological interpretation—are each described in a way that makes comparisons valid and interpretations understandable and repeatable. Furthermore, appropriate reporting of these studies gives other scientists confidence in subsequent analysis of the original data, beyond what is reported in the original manuscript. The aim of developing the JBC checklist for genomics and functional genomics data is to provide a practical tool to help meet these standards without overburdening authors.

## Why a checklist, and why now?

Standards for sharing functional genomics data have existed for years, and community efforts such as MIAME (1) helped establish what “minimum information” should look like. At the same time, even well-established standards can be interpreted differently across fields and technologies, and repositories cannot always enforce completeness computationally. That reality places a practical burden on peer review: editors and reviewers often need to determine whether sufficient information has been supplied. This burden is amplified in cases where authors are unsure of how these data are best reported.

In parallel, the broader movement toward FAIR data principles (2) has sharpened expectations that data and metadata should be findable, accessible, interoperable, and reusable, with clear provenance and workflow transparency. For functional genomics, FAIRness depends heavily on metadata quality, explicit reporting of processing steps, and clarity about the software, statistical methods, and genome references or annotations used. To make these expectations straightforward, consistent, and efficient at submission, JBC has developed a Functional Genomics Data Checklist. The intent is not to create extra bureaucracy, but to help authors present their datasets in a transparent manner that aligns with best practices and facilitates reproduction of the study.

## What JBC expects for functional genomics data?

The checklist focuses on a small set of practical requirements that, when met, substantially improve transparency and reviewer efficiency. While we are implementing them at JBC, we believe these practices should be adopted broadly across journals, as they would strengthen transparency and rigor across the publication ecosystem. This check list ensures authors:

1. Deposit both raw and processed data in established repositories.
2. Provide accession numbers and any required reviewer access information at submission.
3. Provide clear, complete metadata for all samples.
4. Make analysis pipelines and software fully transparent.
5. Report statistical models and tests clearly and justify key choices.
6. Disclose third-party processing and methods.
7. Deposit new code and pipelines with a stable, versioned snapshot.
8. Document key computational dependencies.

Further information on expectations for manuscripts is provided in the full checklist https://legacyfileshare.elsevier.com/promis_misc/jbc-functional-genomics-data-checklist.pdf.

## How the checklist helps authors, reviewers, and readers?

Understanding the data and the conclusions they make is key for the review process and ultimately, for readers and science even more broadly. For manuscripts involving genomic data, the checklist is designed to make this process more efficient for authors (*i.e.*, by reducing guesswork about the data and allowing reviewers and readers to find the data easily). Clear documentation of deposited data also reduces the downstream burden on authors to locate, repackage, or explain datasets long after publication. More broadly, when data are accessible and described properly, they become more discoverable and can be used efficiently by others to build upon an author’s work. This strengthens the value of the work itself and supports the community’s ability to reuse and extend published findings.

## Moving forward

JBC will now include the Functional Genomics Data Checklist as part of the submission process for manuscripts that present functional genomics datasets. We encourage authors to view the checklist as a tool: a concise set of best practices that improves clarity, accelerates review, and increases confidence in the conclusions. Our aim is to ensure that the studies we publish are accompanied by the data access, metadata, and computational transparency needed for rigorous evaluation and meaningful reuse. We anticipate that the Functional Genomics Data Checklist will be an evolving document that will be modified in response to the rapid development of new methods in this area.

## Conflict of interest

The authors declare that they have no conflicts of interest with the contents of this article.
